# Specific reversal agents for direct oral anticoagulants in neurosurgical emergencies – a systematic review

**DOI:** 10.1016/j.rpth.2025.103225

**Published:** 2025-10-13

**Authors:** Laura Zeller, Jonas Rohr, Giovanna Brandi, Christoph Globas, Alexander Kaserer, Matthias Greutmann, Tilman Schubert, Jan-Dirk Studt, Katharina Geiling, Luca Regli, Menno R. Germans

**Affiliations:** 1 Neurocritical Care Unit, Department of Neurosurgery, Institute of Intensive Care Medicine, University Hospital Zurich, University of Zurich, Zurich, Switzerland; 2 Department of Neurosurgery, Kantonsspital Aarau, Aarau, Switzerland; 3 Department of Neurosurgery, University Hospital Zurich, University of Zurich, Zurich, Switzerland; 4 Clinical Neuroscience Center, University Hospital Zurich, University of Zurich, Zurich, Switzerland; 5 Department of Neurology, University Hospital Zurich, University of Zurich, Zurich, Switzerland; 6 Institute of Anesthesiology and Perioperative Medicine, University Hospital Zurich, University of Zurich, Zurich, Switzerland; 7 Department of Cardiology, University Heart Center, University Hospital Zurich, University of Zurich, Zurich, Switzerland; 8 Department for Neuroradiology, University Hospital Zurich, University of Zurich, Zurich, Switzerland; 9 Department of Medical Oncology and Hematology, University Hospital Zurich, University of Zurich, Zurich, Switzerland; 10 Department of Geriatric Medicine and Aging Research, University of Zurich, Zurich, Switzerland

**Keywords:** andexanet alfa, hemostasis in surgery, idarucizumab, neurosurgery, neurosurgical emergencies, oral anticoagulant reversal, perioperative management, specific reversal agent

## Abstract

**Background:**

Direct oral anticoagulants (DOACs) are widely used but pose significant challenges in emergency neurosurgical settings. Idarucizumab and andexanet alfa are approved reversal agents, yet their effectiveness and safety in neurosurgical emergencies remain unclear.

**Objectives:**

This systematic review evaluates the current evidence on DOAC reversal in patients undergoing emergency neurosurgical interventions.

**Methods:**

We conducted a systematic review of case series of a minimum of 5 patients on DOACs receiving andexanet or idarucizumab for emergency neurosurgery (<24 hours after the last DOAC intake). The primary outcome was safety, defined by hemostatic efficacy. Secondary outcomes were thromboembolic events, mortality, and the functional clinical outcome.

**Results:**

Seven studies, comprising 148 patients, met the inclusion criteria. Andexanet was investigated in 4 studies, including 37 patients. Adequate intraoperative hemostasis was confirmed in 80% to 100% of patients. One study showed a thromboembolic event rate of 8.3% and an in-hospital mortality rate of 50% in 12 patients, while others showed lower rates. Idarucizumab was analyzed in 3 studies of 111 patients, of whom 97 were from a single large cohort. Satisfactory hemostasis was reported in 93.5% to 100% of patients. Thromboembolic event rates ranged from 0% to 4.1% and mortality rates were 0%, 3.1%, and 12.5%, respectively.

**Conclusion:**

The use of DOAC reversal agents in emergency neurosurgery appears feasible and may support surgical hemostasis in selected patients. However, further research is warranted, particularly regarding the off-label use of andexanet, given the limited evidence and potential safety concerns.

## Essentials

• Idarucizumab and andexanet alfa can reverse the effects of DOACs in major bleedings.
• These reversal agents show adequate surgical hemostasis in selected patients in neurosurgical emergencies.
• However, available data are very limited and heterogeneous, especially for andexanet alpha.
• More studies are needed to confirm their safety and guide treatment choices.

## **1** Introduction

Intracranial hemorrhage, severe traumatic brain injury, acute hydrocephalus, cerebral edema, or malignant ischemic stroke are the most common neurosurgical emergencies [1]. External ventricular drain (EVD) placement to relieve intracranial pressure, craniotomy for hematoma evacuation, emergency neurovascular surgery, or decompressive craniectomy for malignant stroke are life-saving interventions that must be performed as early as feasible to optimize outcomes. In these situations, the increasing use of direct oral anticoagulants (DOACs) for stroke prevention and venous thromboembolism has introduced new challenges in emergency neurosurgical care.

For example, in intracerebral hemorrhage (ICH), patients treated with DOACs show larger hemorrhage volumes, more frequent intraventricular hemorrhage (IVH), and have increased and prolonged hematoma expansion. This results in higher morbidity and mortality compared with patients not treated with DOACs [2,3]. Furthermore, urgent surgery (including all types of surgeries) in DOAC-treated patients is generally associated with a higher risk of thromboembolism, major bleeding, and higher mortality than elective surgery [[4], [5], [6], [7]]. The management of patients with coagulopathies, especially those induced by the use of DOACs, is challenging.

Direct reversal strategies for DOACs have been lacking until the approval by the European Medicines Agency of idarucizumab in 2015 (Praxbind, Boehringer Ingelheim) and andexanet alfa (AA) in 2019 (Ondexxya, AstraZeneca). Idarucizumab is a monoclonal antibody fragment that reverses the effect of dabigatran. Andexanet alfa is a recombinant protein designed to reverse factor (F)Xa inhibitors and is approved as an antidote for apixaban and rivaroxaban [8,9].

Landmark studies and real-world data have confirmed the hemostatic efficacy of idarucizumab and AA in nonsurgical patients [[10], [11], [12], [13]]. To our knowledge, the safety of their use in patients undergoing emergency neurosurgical procedures has not been systematically investigated.

From a neurosurgical perspective, rapid and effective complete normalization of coagulation is desirable to mitigate potentially serious peri- or postoperative complications, such as persistent or recurrent bleeding or postoperative hemorrhage, all of which can worsen patient outcome. This must be well balanced against thromboembolic risks, including deep venous thrombosis, stroke, or myocardial infarction. With this in mind, the Coagulation Managment in Neurosurgical Diseases (COMAND) project was established to develop optimal coagulation management strategies for several neurosurgical diseases (https://www.comand-project.com) [14]. This systematic review is part of the project and aimed at evaluating the available literature on the effectiveness and clinical outcomes of idarucizumab and AA in patients requiring emergency neurosurgical treatment.

## 2 Methods

This systematic review was performed according to the Cochrane Collaboration guidance and followed the Preferred Reporting Items for Systematic Reviews and Meta-analyses (PRISMA) 2020 statement, including the creation of a flow diagram [15,16].

The review protocol was registered in the International Prospective Register of Systematic Reviews (https://www.crd.york.ac.uk/PROSPERO/ ID: CRD42023473054). The COMAND project has been registered in an open data repository, where additional information is shared with the research community (https://osf.io/preprints/osf/g62wd).

### 2.1 Research question and eligibility criteria

The research question and search were built using the Population/Patient, Intervention, Comparison, Outcome (Time) model. The aim was to study adults (≥18 years) on DOACs who required emergency neurosurgical operations and received a specific DOAC reversal agent, assessing hemostatic effectiveness, postoperative bleeding related to surgery, thromboembolic events, clinical outcomes, and mortality. As an optional control group, patients who received optimal medical care, including prothrombin complex concentrates (PCCs) and massive transfusion protocols, as per local guidelines, were also considered. All types of observational studies and case series with a minimum of 5 cases were considered eligible. Unpublished data were also included.

### 2.2 Search strategy

The search string was developed in close collaboration with a clinical librarian of the University of Zurich. As the number of results was expected to be low and the gray literature could contain relevant documents for the research question, the query was intentionally kept as broad as possible without any language restrictions. We searched the following databases: Embase, Medline, Cochrane Library, Web of Science, and Scopus. The search considered emergency neurosurgical procedures in patients taking DOACs and who received any specific reversal agent, including data that have not yet been peer-reviewed. Regarding the U.S. Food and Drug administration approval of idarucizumab as the first specific reversal agent, a time frame was set from 2015 to the date of the search, February 15, 2024. In addition, we screened oral presentations and abstracts from the following congresses: the International Society on Thrombosis and Haemostasis (ISTH), the American Society of Hematology, the Gesellschaft für Thrombose und Hämostaseforschung, the European Association of Neurosurgical Societies, the American Association of Neurological Surgeons, and the Congress of Neurological Surgeons. As the first included article of the search was published in 2019, congresses from 2019 to December 2023 were considered for abstract screening.

### 2.3 Data management

The search results were conducted across the different databases and merged using EndNote. After deduplication, 2 independent reviewers (L.Z. and J.R.) screened titles and abstracts for the defined eligibility criteria. Discrepancies were resolved by discussion and consensus. In case of inconsistencies despite discussion, M.G. served as a third reviewer.

### 2.4 Study quality

The included studies were subjected to methodological quality assessment by 2 independent reviewers (L.Z. and J.R.) using the National Institutes of Health Quality Assessment Tool, developed by the National Heart, Lung, and Blood Institute, for observational cohort or cross-sectional studies [17]. Each item was rated as yes, no, moderate/unclear, or not applicable. The overall quality appraisal was allocated as follows: good quality (>8 points), intermediate quality (>5 to ≤8 points), or poor quality (≤5 points).

### 2.5 Outcome definitions

Evaluating safety, defined by (intraoperative) effectiveness of hemostasis and/or absence of postoperative bleeding in postoperative imaging, was outlined as the primary outcome. The effectiveness of hemostasis was primarily assessed by the performing surgeon during intraoperative evaluation. Dichotomous evaluation was accepted, along with categories such as “normal,” “mildly abnormal,” “moderately abnormal,” and “severely abnormal.” In cases of EVD placement, postprocedural imaging (typically computed tomography [CT]) was also accepted as proof of adequate hemostasis. Secondary outcomes of interest were the occurrence of thromboembolic events (TEs), such as ischemic stroke, pulmonary embolism, and myocardial infarction, as well as the functional clinical outcome assessed using outcome scales like the modified Rankin Scale, the Glasgow Outcome Scale or the Glasgow Outcome Scale-Extended, and 30-day mortality. Additionally, laboratory assessment of the effectiveness of the reversal agent was evaluated.

## 3 Results

### 3.1 Study selection and characteristics

**Figure 1:**
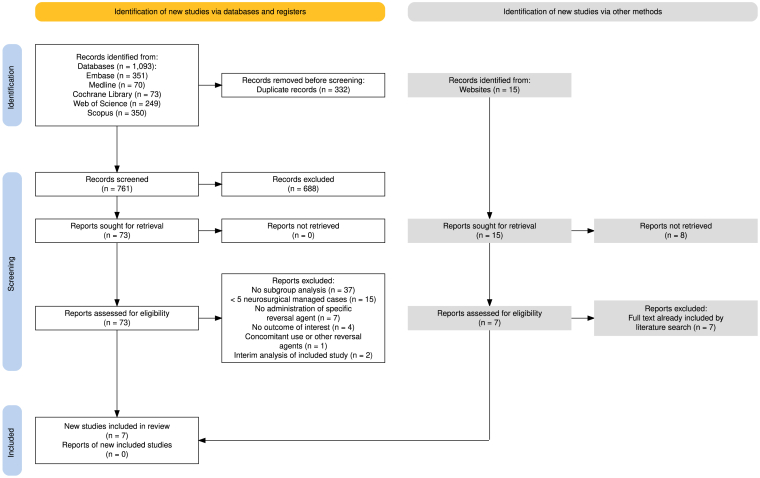
Preferred Reporting Items for Systematic Reviews and Meta-analyses flow diagram illustrating the systematic literature search, screening, eligibility assessment, and final inclusion of studies in the review.

The initial literature search yielded 761 articles after deduplication. After title and abstract screening, the full texts of 9 articles were assessed for eligibility, of which 2 were excluded as they were (interim) analyses of later published data [12,18] (Figure 1). The congress screening revealed 7 relevant documents. Five of those were already included as full texts, whereas 2 articles did not meet the inclusion criteria due to the low number of neurosurgical cases; therefore, no congress document was added to the final analysis (see Supplementary Table S1).

Seven studies were finally included in the systematic review (Figure 1) [15,[19], [20], [21], [22], [23], [24], [25]]. Four studies, with sample sizes ranging from 5 to 15 patients and a total of 37 neurosurgeries performed in 37 patients, reported on the treatment with AA [19,20,23,24]. Three studies reported on idarucizumab and included between 5 and 97 patients in a total of 110 neurosurgical treatments in 110 patients [21,22,25]. No study addressed the full predefined outcome set, highlighting the incomplete nature of the current evidence. None of the articles included a control group.

### 3.2 Quality assessment

**Figure 2:**
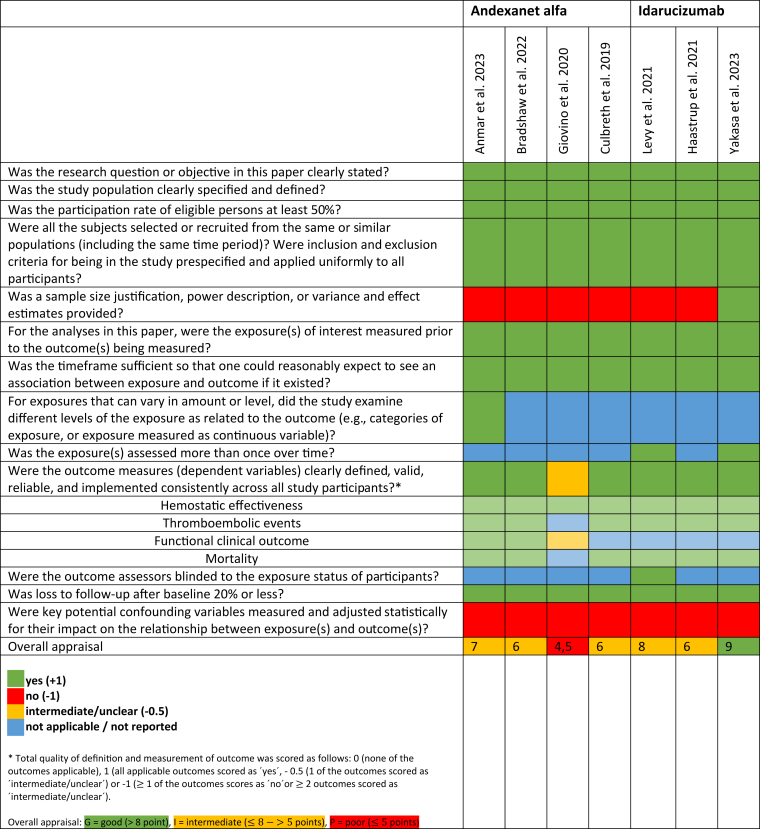
Quality assessment of included studies using the National Institutes of Health Quality Assessment Tool for observational cohort and cross-sectional studies. Overall appraisal scores are provided, with grading as good (G; >8 points), intermediate (I; 6-8 points), or poor (P; <6 points).

The results of the quality assessment are shown in Figure 2. One study was appraised as of good quality, 5 were of intermediate quality, and 1 was of poor quality.

### 3.3 Summary of the included studies

#### 3.3.1 Andexanet alfa

Culbreth et al. [19] were the first to report on patients treated with andexanet prior to an emergency neurosurgery. In a retrospective case series, detailed information on 3 craniotomies for subdural hematoma and 2 EVD placements in ICH was presented. All patients had taken the oral FXa inhibitors within 18 hours prior to presentation. Two EVD placements were performed at the start of the bolus, while 2 craniotomies were performed after completion of the bolus and during the end of the subsequent infusion, and 1 was performed 6 hours after infusion.

Effective hemostasis, as evaluated by the surgeon, was achieved in 4 patients (80%), with a maximum intraoperative blood loss of 50 mL. One surgery required additional blood products, and thus, this was classified as “no effective hemostasis.” No TEs occurred, and 1 patient with intraparenchymal hemorrhage who received an EVD at the time of the andexanet bolus died during the hospitalization, without any information on functional clinical outcome in the other patients. Predose and postdose anti-Xa levels were available for 1 patient and showed adequate reduction after andexanet infusion (4.74 U/mL predose and 1.73 U/mL postdose) [19].

An observational study of 39 anticoagulated patients, published by Giovino et al. [20], reported on 4 craniotomies for subdural hematoma and 1 ventriculostomy after the administration of andexanet. In all cases, good intraoperative hemostasis was reported by the surgeon, and no postoperative rebleeding occurred, as confirmed by a follow-up CT scan at 24 hours. A good functional outcome, without any further specification, was reported for all patients in the craniotomy group. There was no detailed report on TE or mortality for the neurosurgical group. Anti-Xa levels were not presented in the study, while in the whole cohort – including 34 conservatively managed and 5 surgically managed cases – in most of the subjects, the time since the last FXa inhibitor dose was confirmed to be <18 hours, while for 5 patients, it was supposed to be <18 hours [20].

In a single-center, observational, retrospective study including 44 patients receiving andexanet within 24 hours of invasive or surgical procedures, Bradshaw et al. [23] reported on 15 emergency neurosurgical treatments. While the type of surgery was specified (6 craniotomies and 9 EVDs), the underlying pathology was not. Intraoperative hemostatic efficacy was achieved in 12 patients (80%), while the TE rate, in-hospital mortality rate, and 30-day mortality rate for all surgically managed patients (including nonneurosurgical procedures) were 27.3%, 22.7%, and 34.1%, respectively. Anti-Xa assay results were available for 21 patients and were 1.8 U/mL (IQR, 1.4-1.8), drawn 1.1 hours (IQR, 0.5-2.0) prior to bolus, vs 1.4 U/mL (IQR, 0.9-1.8), drawn 5.9 hours (IQR, 1.8-12.1) after infusion completion (*P* = .008). The functional clinical outcome was not assessed, while the median discharge Glasgow Coma Scale (GCS) score of surviving patients with an intracranial event ranged between 10 and 15 points. Postoperative bleeding was not studied [23].

Ammar et al. [24] published data on the placement of 12 EVDs after andexanet administration. The indications were spontaneous ICH with IVH (*n* = 3), IVH (*n* = 3), aneurysmatic subarachnoid hemorrhage with IVH (*n* = 1), hydrocephalus (*n* = 2), traumatic ICH with IVH (*n* = 2), and subarachnoid hemorrhage with hydrocephalus postthrombectomy (*n* = 1). They assessed the safety of EVD placement immediately postandexanet bolus (15 minutes) in 8 patients compared with postandexanet infusion (15 + 120 minutes) in 4 patients by investigating EVD tract hemorrhage or IVH on postoperative imaging. One patient in the postandexanet bolus group had a small, focal, extraaxial collection that required no reoperation and had no further postoperative bleeding. Intraoperative hemostasis was not assessed. The in-hospital mortality rate was 50% (6 patients, equally distributed between the postandexanet bolus group and the postandexanet infusion group). Causes of death were withdrawal of care, multiorgan failure, or global edema with uncontrolled intracranial pressure. There was 1 deep venous thrombosis in the postandexanet bolus group. Pre- and postreversal laboratories for fibrinogen and international normalized ratio were assessed, showing no significant change [24].

In summary, among 37 emergency neurosurgical interventions, andexanet administration led to an intraoperative hemostasis efficacy of 80% to 100%, with TE rates ranging from 0% to 8.3% and 30-day mortality rates ranging from 0% to 50% (see Table). Regarding the type of surgery performed, the available data show no postoperative intra-axial bleedings in 15 EVD placements, while normal intraoperative hemostasis was reported in 6 out of 7 craniotomies (see Supplementary Table S2) [19,20,23,24].

**Table:** Overview of included studies reporting neurosurgical patients receiving anticoagulant reversal with andexanet alfa or idarucizumab.

Study	Neurosurgical patients (*n*)	Type of surgery (*n*)	Hemostatic efficacy^c^ (%)	Postoperative bleedings (*n*)	TE (%)	In-hospital mortality (%)	30-d mortality (%)
Ammar et al. [24]	12	EVD (12)	NA	1	8.3	50	NA
Bradshaw et al. [23]	15^a^	Craniotomy (6), EVD (9)	80^f^	NA	27.3^g^	22.7^g^	34.1^g^
Giovino et al. [20]	5	Craniotomy (4), EVD (1)	100	0	NA	0^d^	NA
Culbreth et al. [19]	5	Craniotomy (2), EVD (2), HE (1)	80	NA	0	20	NA
Levy et al. [22]	8	Craniotomy (8)	100	NA	0	NA	12.5
Haastrup et al. [21]	5	NA	100	NA	0	0	0
Yasaka et al. [25]	97^b^	NA	93.5^f^	NA	4.1^f^	3.1^e^	NA

EVD, external ventricular drain; HE, hematoma evacuation; NA, not available; TE, thromboembolic event.

a The study reports on a total of 20 patients requiring neurological surgery/intervention; 15 patients required cranial neurosurgery.

b A total of 112 patients with neurological surgery/procedure were presented; the supplementary material delivered data for 97 neurosurgically managed patients.

c Definitions varied between studies and percentage described for excellent/good, normal/mildly abnormal, and completion of surgery without report on excessive bleeding.

d Mortality only reported for craniotomy patients, not for EVD.

e Mortality within 5 days after admission of idarucizumab.

f Cohort included neurointerventional and spine procedures.

g Overall cohort included all surgical procedures.

#### 3.3.2 Idarucizumab

Yasaka et al. [25] presented the largest cohort of 112 Japanese patients, of which 97 underwent emergency neurosurgery and 15 underwent endovascular interventions after idarucizumab administration. Adequate intraoperative hemostasis occurred in 93.5% (*n* = 105) of patients. The median time from infusion to intervention was 1.05 hours, and anticoagulation reversal was measured by activated partial thromboplastin time (aPTT) in 26 surgically managed patients, with 23 patients (88.4%) showing an 80% to 100% reduction in aPTT within the first 4 hours after idarucizumab administration. The in-hospital mortality rate was 3.1% for the neurosurgical patients, and TE complications occurred in 4.1% (*n* = 5) of patients. The functional clinical outcome was not reported in the study [25].

Levy et al. published a subgroup analysis of surgically managed patients from the Reversal Effects of Idarucizumab on Active Dabigatran (REVERSE-AD) cohort, reporting on 8 patients who underwent emergency craniotomy after targeted reversal with idarucizumab administration. All patients achieved normal intraoperative hemostasis, without TE, and an in-hospital mortality rate of 12.5% (*n* = 1). The median time to surgery after infusion was 3.3 hours. Of the 3 patients with elevated aPTT, the aPTT was lowered by 100% in 2 patients after idarucizumab infusion. Five patients received additional hemostatic therapy (1 red blood cell, 2 fresh frozen plasma, and 2 tranexamic acid). Functional clinical outcome and postoperative bleeding were not reported in this study.

With the aim of investigating real-world experience with idarucizumab, Haastrup et al. [21] reported 46 patients treated with idarucizumab in clinical practice. Five of them underwent unspecified neurosurgical interventions. Effective intraoperative hemostasis was achieved in all patients without TE complications or death at 30 days. Preoperative dabigatran levels were presented for 26 patients; hence, there was no assessment postinfusion. Postoperative bleeding and functional clinical outcome were not reported [21].

In summary, the administration of idarucizumab demonstrated intraoperative hemostasis efficacy of 93.5% to 100%, with TE rates ranging from 0% to 4.1% and 30-day mortality rates from 0% to 12.5% in emergency neurosurgical patients (see Table). Importantly, only Levy et al. specified the hemostatic results in relation to the type of surgery (see Supplementary Table S2).

## 4 Discussion

The primary aim of this review was to provide an overview of the published literature covering the administration and outcomes of andexanet and idarucizumab in the context of emergency neurosurgical procedures.

The primary outcomes of interest were hemostatic effectiveness and/or absence of postoperative bleeding. Both agents, particularly idarucizumab, achieved high rates of effective intraoperative hemostasis (≥ 80% for andexanet and ≥ 93.5% for idarucizumab) without clinically relevant postoperative bleeding. Due to the minimally invasive nature of EVD placement, direct intraoperative assessment of hemostasis is often not feasible; thus, postoperative imaging may serve as the primary surrogate. For andexanet, a postprocedural CT image was used to confirm the absence of bleeding in most cases. However, follow-up imaging was not included in all reports, which represents a limitation of the available data. For idarucizumab, the majority of available data originate from the cohort reported by Yasaka et al., who did not specify the type of surgical procedure performed [25].

In terms of the laboratory assessment for screening and quantitative assessment of the dabigatran effect, the REVERSE-AD study demonstrated complete reversal of serum dabigatran levels and restoration of normal diluted thrombin time (TT) and aPTT *in vivo* as early as 10 to 30 minutes after the infusion of 5 g intravenous idarucizumab, given as two 2.5 g bolus infusions, and lasting up to 24 hours. The recommended screening assays for dabigatran are aPTT and/or TT, while diluted TT and direct thrombin inhibitor assay are used for quantitative assessment [26]. Importantly, a normal aPTT excludes above-therapy dabigatran levels, but does not rule out the presence of dabigatran in the therapeutic range [27,28]. In the presented data, Yasaka et al. [25] showed an 80% to 100% reduction in aPTT within the first 4 hours in 23 of 26 patients but did not assess TT. The aPTT was elevated in only 3 of 8 patients presented by Levy et al. [22] and decreased by 100% after idarucizumab in 2 of these 3 patients.

The a Novel Antidote to the Anticoagulation Effects of Factor Xa Inhibitors (ANNEXA-4) study, a multicenter, prospective, phase 3b/4, single-group cohort study, showed a reduction in anti-Xa levels of up to 94% immediately after the administration of andexanet in patients with prespecified baseline anti-Xa activity levels above a threshold of >75 ng/mL for apixaban and rivaroxaban. Importantly, this effect diminished to a 35.6% to 38% reduction for apixaban and 43.2% to 61% for rivaroxaban at 4 to 12 hours after application [11]. Among the data included in our review, Bradshaw et al. [23] showed a reduction in anti-Xa levels from 1.8 U/mL to 1.4 U/mL after AA in 21 patients, while Culbreth et al. [19] showed an adequate reduction in anti-Xa levels in 1 patient. It is important to remember that routine anti-Xa assays underestimate the efficacy of reversal, while a modified anti-Xa assay using a lower sample predilution gives more accurate information [29].

Rapid restoration of coagulation is required in emergency neurosurgery and is provided by both agents. However, in prolonged or delayed (>4 hours after administration) neurosurgical procedures, the potential for recurrence must be considered, especially when using andexanet.

The TE rates for idarucizumab, up to 4.1% in the largest cohort (*N* = 97), which is the most representative, are well in line with the TE rate of 4.8% reported in REVERSE-AD [11,30,31]. The reported TE rates, ranging from 0% to 8.3% for neurosurgical managed patients after AA, are lower than the 14% reported in a systematic review and meta-analysis for conservatively managed patients with ICH treated with AA [32]. However, these TE rates for AA were derived from small cohorts (5-12 patients) and are therefore not significant. A recent randomized controlled trial confirmed higher TE rates with andexanet in conservatively managed ICH management (10.3% AA vs 5.6% usual care) [33].

Clinical outcome was assessed in only 2 of the andexanet trials and in none of the idarucizumab trials. While the patients in the cohort of Ammar et al. [24] were more severely impaired or died (modified Rankin Scale 4-6), Bradshaw et al. [23] reported a favorable outcome with a GCS score of up to 15 points in survivors. Admission GCS scores were much higher in the Bradshaw et al. [23] cohort (median, 10 vs 6), which, along with the underlying pathologies themselves and several other confounding factors, limits comparability. Importantly, GCS scores do not sufficiently reflect the functional status of the patient, thus highlighting the need for more comprehensive outcome measures in the future.

Mortality rates varied widely (0%-50% in AA and 0%-12.5% in idarucizumab), reflecting the low case numbers, lack of statistical power, heterogeneous pathology, and study designs. In the largest cohort of 97 patients by Yasaka et al., the mortality rate was 3.1%, while Levy et al. reported a 30-day mortality rate of 1 in 8 patients (12.5%) who underwent a craniotomy. Excluding Ammar et al.'s [24] study, the mortality rates of all other studies for both agents are lower than the recently published 90-day mortality rates of 35% to 37% in a mixed ICH cohort on DOACs who did not receive a specific reversal agent but were rather treated without hemostatic agents or with PCCs [34].

For andexanet, none of the studies were rated “good” according to the National Institutes of Health Quality Assessment Tool. A total of 37 patients were studied, with a maximum sample size of 15 patients, which limits the interpretation of the results. For idarucizumab, most of the data stem from a single cohort. While the quality of outcome reporting in this study is good, the lack of information on the underlying pathology or type of surgery performed limits the interpretability and generalizability of its findings, particularly with regard to intraoperative hemostasis. To date, only idarucizumab is approved for use in surgically treated patients [35]. However, guidelines recommend the use of andexanet as an adjunct to necessary endoscopy, angiography, or surgery under predefined conditions [10,[36], [37], [38]].

Beyond specific reversal agents, 4-factor PCCs (4F-PCCs) are often applied in clinical practice for the management of DOAC-associated bleeding. Despite being off-label, 4F-PCCs are more widely available and generally more cost-effective compared with andexanet. The recently published ISTH guidance highlights the growing role of PCCs in DOAC reversal, especially in settings where specific agents are unavailable or contraindicated [39]. A recent systematic review and meta-analysis by Chaudhary et al. [32] compared reversal agents in intracranial hemorrhage and found similar anticoagulation reversal rates among idarucizumab (82%), andexanet (75%), and 4F-PCC (77%). Notably, all-cause mortality was lowest in the idarucizumab group (11%) compared with 24% for andexanet and 26% for PCC [32]. Reported TE rates were 5% (idarucizumab), 8% (PCC), and 14% (andexanet). While the study was not limited to neurosurgical patients, these data underscore the need for further direct comparisons in neurosurgical emergency settings. The Andexanet for Factor Xa Inhibitor-Associated Acute Intracerebral Hemorrhage (ANNEXA-I) trial included a “usual care” arm with PCC use, highlighting that andexanet significantly reduced hematoma expansion compared with usual care but was associated with an increased ischemic stroke risk (6.5% vs 1.5%) [33]. However, patients requiring surgery within 12 hours were excluded, limiting the applicability to emergency neurosurgery.

Future prospective studies comparing specific and nonspecific reversal strategies (eg, 4F-PCCs) in neurosurgical emergencies are critically needed to inform evidence-based guidelines.

## 5 Conclusion and Limitations

In summary, the use of DOAC reversal agents in emergency neurosurgery appears feasible and may support surgical hemostasis in selected patients. However, further research is warranted, particularly regarding the off-label use of andexanet, given the limited evidence and potential safety concerns. Key confounding factors- such as the underlying pathology (eg, traumatic vs spontaneous hemorrhage), type of surgical procedure, concurrent blood product administration, and patient-specific characteristics-should be consistently considered. Ultimately, prospective systematic studies with clearly defined outcome measures are needed to guide evidence-based decision-making in this high-risk population.

## Declaration of AI and AI-Assisted Technologies in the Writing Process

During the preparation of this work the authors used DeepL Write and ChatGPT (OpenAI, GPT-4) in order to refine the language and improve clarity. After using this tool/service, the authors reviewed and edited the content as needed and take full responsibility for the content of the publication.
