# Supplementary material for: Specific reversal agents for direct oral anticoagulants in neurosurgical emergencies – a systematic review
Source: Res Pract Thromb Haemost. 2025 Oct 13;9(8):103225. doi: 10.1016/j.rpth.2025.103225 (PMC12663499; doi:10.1016/j.rpth.2025.103225)
Supplement: Supplementary Table 1 [file mmc1.docx]

Table 1, Overview of the congress screening
