# Supplementary material for: Specific reversal agents for direct oral anticoagulants in neurosurgical emergencies – a systematic review
Source: Res Pract Thromb Haemost. 2025 Oct 13;9(8):103225. doi: 10.1016/j.rpth.2025.103225 (PMC12663499; doi:10.1016/j.rpth.2025.103225)
Supplement: Supplementary Table 2 [file mmc2.docx]

Table 2, Hemostasis by type of surgery

| Type of surgery* | Patients (n) | Intraoperative hemostasis achieved (n/n) | Postoperative bleedings | Study |
| --- | --- | --- | --- | --- |
| **Andexanet alfa** |  |  |  |  |
| EVD | 15 | 3/3 *** | No postoperative intraaxial bleeding in CT-scan | Giovino et al. 2020; Ammar et al. 2023 |
| Craniotomy (intraaxial) | 1 | 1/1 | NA | Culbreth et al., 2019 |
| Craniotomy (extraaxial) | 6 | 5/6 | No postoperative bleeding in CT-scan** | Culbreth et al., 2019; Giovino et al., 2020 |
| **Idarucizumab** |  |  |  |  |
| Craniotomy (unspecified) | 8 | 8/8 | NA | Levy et al., 2021 |
| Note: EVD = External Ventricular Drain; NA = Not Available. Some values refer to subgroups or were approximated from narrative descriptions.  * Data from Bradshaw et al., Haastrup et al. and Yasaka et al. are not presented as hemostatic efficacy is not presented by type of surgery in those studies  ** Data only available in 4 cases published by Giovino et al.  *** Ammar et al. did not report on intraoperative hemostasis | | | | |
